# Sex difference in the associations among hyperuricemia with self-reported peptic ulcer disease in a large Taiwanese population study

**DOI:** 10.3389/fmed.2024.1383290

**Published:** 2024-06-10

**Authors:** Chi-Sheng Yang, Jiun-Hung Geng, Pei-Yu Wu, Jiun-Chi Huang, Huang-Ming Hu, Szu-Chia Chen, Chao-Hung Kuo

**Affiliations:** ^1^Department of Internal Medicine, Kaohsiung Medical University Hospital, Kaohsiung Medical University, Kaohsiung, Taiwan; ^2^Division of Gastroenterology, Department of Internal Medicine, Kaohsiung Medical University Hospital, Kaohsiung Medical University, Kaohsiung, Taiwan; ^3^Department of Urology, Kaohsiung Municipal Siaogang Hospital, Kaohsiung Medical University Hospital, Kaohsiung Medical University, Kaohsiung, Taiwan; ^4^Department of Urology, Kaohsiung Medical University Hospital, Kaohsiung Medical University, Kaohsiung, Taiwan; ^5^Department of Internal Medicine, Kaohsiung Municipal Siaogang Hospital, Kaohsiung Medical University Hospital, Kaohsiung Medical University, Kaohsiung, Taiwan; ^6^Division of Nephrology, Department of Internal Medicine, Kaohsiung Medical University Hospital, Kaohsiung Medical University, Kaohsiung, Taiwan; ^7^Faculty of Medicine, College of Medicine, Kaohsiung Medical University, Kaohsiung, Taiwan

**Keywords:** sex difference, hyperuricemia, self-reported peptic ulcer disease, Taiwan Biobank, Risk factors

## Abstract

**Background:**

Hyperuricemia may play a role in various systemic diseases. However, few studies have investigated the relationship between hyperuricemia and the risk of peptic ulcer disease (PUD). Therefore, in this population-based study, we enrolled over 120,000 participants from the Taiwan Biobank (TWB) and examined the risk factors for self-reported PUD. In addition, we investigated sex differences in the association between hyperuricemia and self-reported PUD.

**Methods:**

Data of 121,583 participants were obtained from the TWB. Male participants with a serum uric acid level >7 mg/dl and female participants with a serum uric acid level >6 mg/dl were classified as having hyperuricemia. Details of self-reported PUD were obtained by questionnaire. The association between hyperuricemia and self-reported PUD in the male and female participants was examined using multivariable logistic regression analysis.

**Results:**

The overall prevalence of self-reported PUD was 14.6%, with a higher incidence in males (16.5%) compared to females (13.5%). After multivariable adjustment, male sex [vs. female sex; odds ratio (OR) = 1.139; 95% confidence interval (CI) = 1.084–1.198; *p* < 0.001], and hyperuricemia (OR = 0.919; 95% CI = 0.879–0.961; *p* < 0.001) were significantly associated with self-reported PUD. Further, a significant interaction was found between sex and hyperuricemia on self-reported PUD (*p* = 0.004). Hyperuricemia was associated with a low risk of self-reported PUD in males (OR = 0.890; 95% CI = 0.837–0.947; *p* < 0.001) but not in females (*p* = 0.139).

**Conclusion:**

The prevalence of self-reported PUD was higher in the male participants than in the female participants. Hyperuricemia was associated with low prevalence of self-reported PUD in males, but not in females. Further studies are needed to clarify the mechanisms behind these observations and verify the potential protective role of hyperuricemia on the development of self-reported PUD.

## Introduction

Peptic ulcer disease (PUD) is defined as a mucosa defect greater than 3–5 mm extending through the muscularis mucosa over the gastrointestinal tract, although it usually presents in the stomach and proximal duodenum (1). A 2020 study, which analyzed PUD burden according to the Global Burden of Disease, Injuries and Risk Factors Study, estimated that there were 8.09 million prevalent cases in 2019 globally (2). A 2004 prospective study in Taiwan of 6,457 subjects who underwent esophagogastroduodenoscopy during a health examination found that two-thirds of the patients diagnosed with PUD endoscopically were asymptomatic (3). PUD is associated with Helicobacter pylori infection (4) and the use of nonsteroidal anti-inflammatory drugs (NSAIDs) (5), and additional risk factors include advanced age, smoking, and alcoholism (6, 7). These factors may influence various aspects of gastrointestinal physiology, such as gastric acid secretion and mucosal defense mechanisms, ultimately contributing to the development of PUD (8). Sex differences have been observed in many diseases, including cancer, liver and cardiovascular diseases, and these differences can have an essential influence on the clinical presentation, disease progression, and response to treatment (9). While PUD was predominantly observed in males in the past, there has been a shift from a male-dominated pattern in Western countries to a nearly equal prevalence between males and females (10). PUD can lead to serious complications such as acute upper gastrointestinal bleeding, perforation, gastric outlet obstruction, and even mortality (11). Therefore, identifying risk factors which may be associated with PUD is important to decrease healthcare system burden and prompt the development of new treatment strategies for improving patient care.

Hyperuricemia is a chronic disease caused by high levels of uric acid due to conditions including dysfunctional purine metabolism and a reduction in the excretion of uric acid (12, 13). Purine metabolism to uric acid occurs through the catalyzation of hypoxanthine to xanthine by xanthine oxidase (14). Moreover, the reactive oxygen species generated during this process have been shown to contribute to metabolic dysfunction (14). These mechanisms imply that hyperuricemia may play a role in various systemic diseases. Associations between hyperuricemia and a higher risk of gout (15) and cardiovascular issue such as hypertension, low left ventricular ejection fraction, and high left atrial diameter have been reported (16, 17), along with dyslipidemia, thyroid dysfunction, chronic kidney disease, and metabolic syndrome (15, 16, 18, 19). However, several studies have suggested that hyperuricemia or gout might act as a protective factor against neurodegenerative diseases such as Alzheimer’s disease or neurological functional outcomes after an acute ischemic stroke (20–22). Nevertheless, some studies have reported conflicting results (23), and the same debate has arisen in the context of osteoporosis and hyperuricemia. Some studies have indicated that individuals with normal or elevated levels of uric acid were associated with a decrease in bone mineral density and lower risk of bone fractures (24). Other studies have demonstrated that elevated levels of serum uric acid were linked to increased bone mass, reduced bone turnover, and a lower incidence of vertebral fractures in postmenopausal women (25). Recent studies have reported that the intestine may play a crucial role in the excretion of uric acid outside the kidneys (26). However, few studies have investigated the relationship between hyperuricemia and the risk of PUD. Therefore, in this population-based study, we enrolled over 120,000 participants from the Taiwan Biobank (TWB) and examined the risk factors for self-reported PUD. In addition, we investigated sex differences in the association between hyperuricemia and self-reported PUD.

## Materials and methods

### TWB

To enhance biomedical and epidemiological research and address the aging population in Taiwan, the TWB is an ongoing prospective study launched by the Ministry of Health and Welfare in 2012 of community-dwelling cancer-free women and men (27, 28). Ethical approval for the TWB was given by the Ethics and Governance Council of the TWB and Institutional Review Board on Biomedical Science Research, Academia Sinica, Taiwan.

The TWB contains medical, genomic and lifestyle factor data, including age, weight, height, and diagnoses of hypertension and diabetes mellitus (DM). In addition, laboratory tests on fasting serum samples (Roche Diagnostics GmbH, D-68298 Mannheim COBAS Integra 400) are conducted to collect data on glucose, hemoglobin, triglycerides, total cholesterol, high- and low-density lipoprotein cholesterol (HDL-C/LDL-C), and uric acid. Estimated glomerular filtration rate (eGFR) and serum creatinine levels were calculated as reported in previous studies (29).

The average of three blood pressure measurements was used for analysis, with each measurement being performed in the absence of caffeine, nicotine and exercise by a nurse using an electronic monitor. Regular exercise was defined according to the “Physical Fitness 333 Plan” in Taiwan as at least three sessions of exercise per week with each session lasting at least 30 min (30). This study complies with the Declaration of Helsinki and was performed according to institutional review board approval (KMUHIRB-E(I)-20210058).

### Sample population and sample size

**FIGURE 1:**
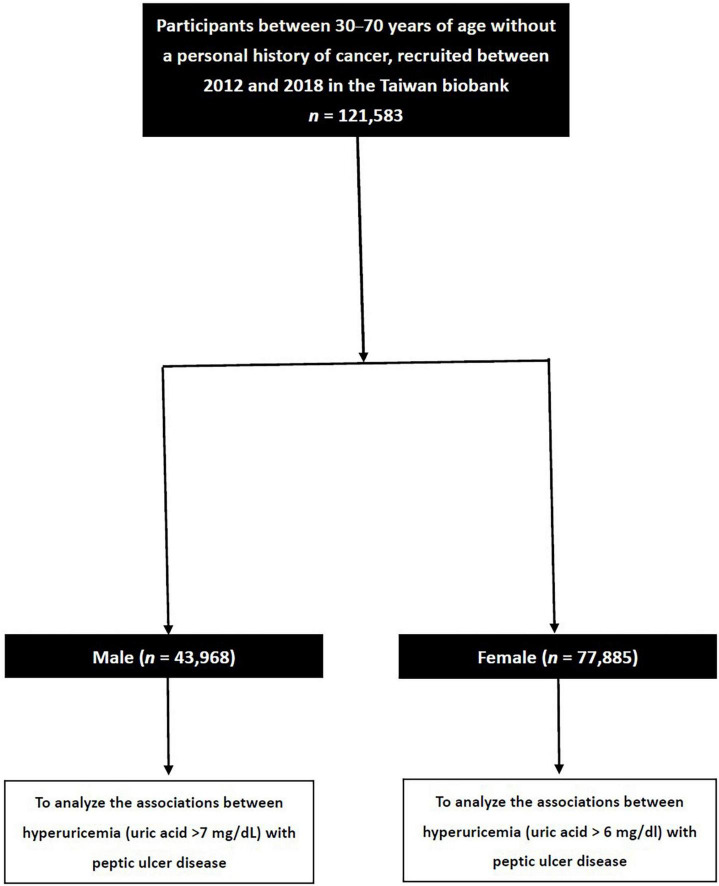
Flowchart of study population.

The TWB enrolls cancer-free members of the community aged 30–70 years, and includes data on medical, genetic, and lifestyle factors. We collected 121,583 enrollees in the TWB. These participants were then classified into those with and without hyperuricemia based on a serum uric acid concentration of >7.0 and >6.0 mg/dl in males and females, respectively (31) (Figure 1).

### Definitions of self-reported PUD

A history of self-reported PUD was recorded using self-reported questionnaires. The presence of self-reported PUD was defined by asking the participants whether they a history of PUD.

### Statistical analysis

The statistical analyses in this study were performed using SPSS version 19.0 for Windows (IBM Inc., Armonk, NY, USA). Continuous variables were expressed as mean ± standard deviation, and between-group differences were analyzed using the independent *t*-test. Categorical variables were presented as frequencies and percentages, and between-group differences were analyzed using the Chi-square test. Associations between hyperuricemia and self-reported PUD in the male and female participants were examined using multivariable logistic regression analysis, which included significant variables in univariable analysis. An interaction *p* in logistic analysis: Model disease (y) = x1 + x2 + x1 × x2 + covariates. x1 × x2 was the interaction term, in which y = self-reported PUD; x1 = sex; x2 = hyperuricemia; covariates = age, sex, DM, hypertension, smoking and alcohol history, regular exercise habit, systolic blood pressure (SBP), body mass index (BMI), hyperuricemia, fasting glucose, hemoglobin, triglycerides, total cholesterol, LDL-cholesterol, and eGFR. A two-tailed *p*-value < 0.05 was considered statistically significant.

## Results

Of the 121,583 participants, 43,698 were male and 77,885 were female, with a mean age of 49.9 ± 11.0 years. The overall prevalence of self-reported PUD in the study cohort was 14.6%, and the difference between the male and female participants was significant (16.5% vs. 13.5%, *p* < 0.001).

### Comparison of the participants with and without self-reported PUD

Table 1 shows the comparisons of the participants with and without self-reported PUD. The participants in the self-reported PUD group were older, predominantly female, and had higher rates of DM, hypertension, smoking and alcohol consumption, regular exercise, and higher SBP, uric acid, hemoglobin, triglycerides, total cholesterol, and LDL-C, and lower BMI, eGFR and fasting glucose compared to those without self-reported PUD (Table 1).

**TABLE 1:** Clinical characteristics of the study participants classified by the presence of self-reported PUD.

Characteristics	Self-reported PUD (*n* = 121,583)		
	Self-reported PUD (−) (*n* = 103,887)	Self-reported PUD (+) (*n* = 17,696)	*p*
Age (year)	49.3 ± 11.0	53.2 ± 1.01	<0.001
Male sex (%)	35.1	40.7	<0.001
DM (%)	4.9	6.7	<0.001
Hypertension (%)	11.7	15.6	<0.001
Smoking history (%)	26.4	32.6	<0.001
Alcohol history (%)	8.2	10.3	<0.001
Regular exercise habits (%)	39.9	44.5	<0.001
Systolic BP (mmHg)	120.3 ± 18.7	121.1 ± 18.3	<0.001
Diastolic BP (mmHg)	73.8 ± 11.4	73.9 ± 11.1	0.076
BMI (kg/m^2^)	24.2 ± 3.8	24.1 ± 3.7	<0.001
**Laboratory parameters**			
Uric acid (mg/dl)	5.42 ± 1.43	5.46 ± 1.40	<0.001
Hyperuricemia (%)	19.5%	19.0%	0.199
Fasting glucose (mg/dl)	95.8 ± 20.8	86.8 ± 19.9	<0.001
Hemoglobin (g/dl)	13.7 ± 1.6	13.9 ± 1.6	<0.001
Triglyceride (mg/dl)	115.2 ± 94.8	117.8 ± 89.0	0.001
Total cholesterol (mg/dl)	195.5 ± 35.9	196.6 ± 35.6	<0.001
HDL-C (mg/dl)	54.6 ± 13.4	54.4 ± 13.7	0.111
LDL-C (mg/dl)	120.8 ± 31.8	121.5 ± 31.6	0.007
eGFR (ml/min/1.73 m^2^)	103.8 ± 23.9	100.4 ± 23.6	<0.001

Hyperuricemia was defined as serum uric acid concentration greater than 7.0 and 6.0 mg/dl in the male and female participants, respectively. PUD, peptic ulcer disease; DM, diabetes mellitus; BP, blood pressure; BMI, body mass index; HDL-C, high-density lipoprotein cholesterol; LDL-C, low-density lipoprotein cholesterol; eGFR, estimated glomerular filtration rate.

### Factors associated with self-reported PUD

Table 2 shows the factors associated with self-reported PUD. The results of multivariable logistic regression analysis with adjustments for the covariates listed in section “Statistical analysis” showed that older age (*p* < 0.001), male sex [vs. female sex; odds ratio (OR) = 1.139, 95% confidence interval (CI) = 1.084–1.198, *p* < 0.001], DM (*p* = 0.001), hypertension (*p* < 0.001), smoking history (*p* < 0.001), alcohol history (*p* < 0.001), without regular exercise habits (*p* < 0.001), low SBP (*p* < 0.001), low BMI (*p* < 0.001), without hyperuricemia (OR = 0.919; 95% CI = 0.879–0.961, *p* < 0.001), low fasting glucose (*p* < 0.001), and high hemoglobin (*p* = 0.019) were significantly associated with self-reported PUD (Table 2).

**TABLE 2:** Determinants for self-reported PUD using multivariable logistic regression analysis.

Variables	Multivariable (self-reported PUD)	
	Odds ratio (95% CI)	*p*
Age (per 1 year)	1.038 (1.036–1.040)	<0.001
Male (vs. female)	1.139 (1.084–1.198)	<0.001
DM	1.142 (1.058–1.273)	0.001
Hypertension	1.160 (1.103–1.220)	<0.001
Smoking history	1.286 (1.232–1.342)	<0.001
Alcohol history	1.113 (1.051–1.179)	<0.001
Regular exercise habits	0.939 (0.907–0.972)	<0.001
Systolic BP (per 1 mmHg)	0.993 (0.992–0.994)	<0.001
BMI (per 1 kg/m^2^)	0.982 (0.977–0.987)	<0.001
**Laboratory parameters**		
Hyperuricemia	0.919 (0.879–0.961)	<0.001
Fasting glucose (per 1 mg/dl)	0.998 (0.997–0.999)	<0.001
Hemoglobin (per 1 g/dl)	1.016 (1.003–1.030)	0.019
Triglyceride (per 10 mg/dl)	1.002 (1.000–1.004)	0.109
Total cholesterol (per 10 mg/dl)	0.993 (0.982–1.004)	0.191
LDL-C (per 1 mg/dl)	1.001 (1.000–1.002)	0.143
eGFR (per 1 ml/min/1.73 m^2^)	0.999 (0.999–1.000)	0.099

Values expressed as odds ratio and 95% confidence interval (CI). Abbreviations are the same as in Table 1. Adjusted for age, sex, DM and hypertension, smoking and alcohol history, regular exercise habit, systolic BP, BMI, uric acid, fasting glucose, hemoglobin, triglyceride, total cholesterol, LDL-cholesterol, and eGFR. Hyperuricemia was defined as serum uric acid concentration greater than 7.0 and 6.0 mg/dl in the male and female participants, respectively.

### Comparisons of the male and female participants with and without self-reported PUD

Table 3 shows the comparisons of the male and female participants with and without self-reported PUD. The male participants with self-reported PUD were older and had higher rates of DM, hypertension, smoking and alcohol consumption, regular exercise, and higher HDL-C, and lower diastolic blood pressure, BMI, uric acid, hyperuricemia prevalence, hemoglobin, triglycerides, LDL-C, and eGFR than those without self-reported PUD (Table 3). The female participants with self-reported PUD were older and had higher prevalence rates of DM, hypertension, alcohol and smoking consumption, regular exercise, and higher SBP, uric acid, fasting glucose, hemoglobin, triglyceride, total cholesterol, and LDL-C, and lower BMI and eGFR than those without self-reported PUD (Table 1).

**TABLE 3:** Clinical characteristics of the study participants classified by the presence of different sex and self-reported PUD.

Characteristics	Male (*n* = 43,698)			Female (*n* = 77,885)		
	Self-reported PUD (−) (*n* = 36,494)	Self-reported PUD (+) (*n* = 7,204)	*p*	Self-reported PUD (−) (*n* = 67,393)	Self-reported PUD (+) (*n* = 10,492)	*p*
Age (year)	49.1 ± 11.4	53.8 ± 10.2	<0.001	49.4 ± 10.8	52.7 ± 9.9	<0.001
DM (%)	6.5	8.4	<0.001	4.0	5.6	<0.001
Hypertension (%)	16.0	21.1	<0.001	9.3	11.9	<0.001
Smoking history (%)	56.3	62.8	<0.001	10.1	11.8	<0.001
Alcohol history (%)	18.2	21.4	<0.001	2.8	3.2	0.017
Regular exercise habits (%)	41.5	46.6	<0.001	39.0	43.1	<0.001
Systolic BP (mmHg)	126.3 ± 17.3	126.7 ± 17.2	0.098	117.1 ± 18.6	117.5 ± 18.1	0.017
Diastolic BP (mmHg)	78.5 ± 11.1	78.2 ± 10.7	0.024	71.2 ± 10.8	71.0 ± 10.4	0.069
BMI (kg/m^2^)	25.5 ± 3.6	24.9 ± 3.5	<0.001	23.6 ± 3.8	23.5 ± 3.7	0.028
**Laboratory parameters**						
Uric acid (mg/dl)	6.45 ± 1.37	6.28 ± 1.35	<0.001	4.86 ± 1.12	4.90 ± 1.13	0.002
Hyperuricemia (%)	30.5	26.2	<0.001	13.5	14.2	0.051
Fasting glucose (mg/dl)	99.3 ± 23.6	99.8 ± 22.2	0.064	93.9 ± 18.9	94.8 ± 17.9	<0.001
Hemoglobin (g/dl)	15.1 ± 1.2	15.0 ± 1.3	<0.001	13.0 ± 1.3	13.1 ± 1.2	<0.001
Triglyceride (mg/dl)	138.8 ± 121.4	133.0 ± 97.7	<0.001	102.5 ± 73.6	107.3 ± 80.8	<0.001
Total cholesterol (mg/dl)	192.0 ± 35.2	191.2 ± 34.5	0.080	197.4 ± 36.1	200.2 ± 35.8	<0.001
HDL-C (mg/dl)	47.9 ± 11.1	48.5 ± 11.3	<0.001	58.2 ± 13.2	58.5 ± 13.7	0.093
LDL-C (mg/dl)	121.9 ± 31.5	120.9 ± 31.4	0.020	120.3 ± 31.9	121.9 ± 31.7	<0.001
eGFR (ml/min/1.73 m^2^)	94.1 ± 19.3	92.0 ± 20.0	<0.001	109.0 ± 24.5	106.2 ± 24.1	<0.001

Abbreviations are the same as in Table 1. Hyperuricemia was defined as serum uric acid concentration greater than 7.0 and 6.0 mg/dl in the male and female participants, respectively.

### Association and interaction of hyperuricemia with self-reported PUD in the male and female participants

Table 4 shows the association and interaction of hyperuricemia with self-reported PUD in the male and female participants. The results of multivariable logistic regression analysis with adjustments for the covariates listed in section “Statistical analysis” except sex showed that hyperuricemia was associated with a lower risk of self-reported PUD (OR = 0.890; 95% CI = 0.837–0.947; *p* < 0.001) in the male participants, but that hyperuricemia was not associated with self-reported PUD (OR = 0.952; 95% CI = 0.893–1.016; *p* = 0.139) in the female participants. In addition, a significant interaction was found between sex and hyperuricemia on self-reported PUD (*p* = 0.004) (Table 4).

**TABLE 4:** Association of factors with self-reported PUD in different sex using multivariable logistic regression analysis.

Characteristics	Male (*n* = 43,698)			Female (*n* = 77,885)		
	OR	95% CI	*p*	OR	95% CI	*p*
Age (per 1 year)	1.039	1.036–1.042	<0.001	1.037	1.034–1.039	<0.001
DM	1.047	0.938–1.170	0.414	1.237	1.112–1.376	<0.001
Hypertension	1.183	1.102–1.270	<0.001	1.140	1.060–1.225	<0.001
Smoking history	1.226	1.161–1.295	<0.001	1.387	1.296–1.483	<0.001
Alcohol history	1.135	1.062–1.212	<0.001	1.084	0.959–1.225	0.197
Regular exercise habits	0.928	0.879–0.980	0.008	0.949	0.907–0.992	0.022
Systolic BP (per 1 mmHg)	0.995	0.993–0.996	<0.001	0.993	0.991–0.994	<0.001
BMI (per 1 kg/m^2^)	0.968	0.960–0.977	<0.001	0.990	0.984–0.997	0.004
**Laboratory parameters**						
Hyperuricemia (%)	0.890	0.837–0.947	<0.001	0.952	0.893–1.016	0.139
Fasting glucose (per 1 mg/dl)	0.998	0.997–0.999	0.005	0.998	0.997–0.999	0.005
Hemoglobin (per 1 g/dl)	1.007	0.984–1.029	0.565	1.026	1.008–1.044	0.004
Triglyceride (per 10 mg/dl)	1.000	0.997–1.003	0.911	1.004	1.001–1007	0.005
Total cholesterol (per 10 mg/dl)	0.995	0.976–1.014	0.624	0.995	0.981–1.008	0.432
LDL-C (per 1 mg/dl)	1.001	0.999–1.003	0.451	1.001	0.999–1.002	0.468
eGFR (per 1 ml/min/1.73 m^2^)	0.999	0.998–1.001	0.435	0.999	0.998–1.000	0.123

Values expressed as odds ratio (OR) and 95% confidence interval (CI). Abbreviations are the same as in Table 1. Adjusted for age, DM and hypertension, smoking and alcohol history, regular exercise habit, systolic BP, BMI, hyperuricemia, fasting glucose, hemoglobin, triglyceride, total cholesterol, LDL-cholesterol, and eGFR. Hyperuricemia was defined as serum uric acid concentration greater than 7.0 and 6.0 mg/dl in the male and female participants, respectively.

## Discussion

In this large Taiwanese population-based study, we examined the risk factors for self-reported PUD and sex differences in the association between hyperuricemia and self-reported PUD. Our results showed that compared to the female participants, the male participants had a higher prevalence of self-reported PUD. Further, a significant interaction was found between sex and hyperuricemia on self-reported PUD, and hyperuricemia was associated with a low prevalence of self-reported PUD in the male participants, but not in the female participants.

Our finding of a higher prevalence of self-reported PUD in males compared to females (16.5% vs. 13.5%, *p* < 0.001), male sex (vs. female sex; OR = 1.139; 95% CI = 1.084–1.198; *p* < 0.001) was significantly associated with self-reported PUD, which is similar to a large-scale, multicenter trial involving over 2,000 patients taking NSAIDs (32). The trial aimed to assess the effectiveness of omeprazole compared to other agents including misoprostol and ranitidine in healing and preventing NSAID-induced ulcers, and the results showed that the incidence of these lesions tended to be higher in in men (62%) compared to women (50%) (32). In addition, duodenal ulcers were more prevalent in men (26%) than in women (8%) (32). In a population-based study involving 204 countries, males had a higher incidence and prevalence of PUD, along with higher mortality and disability-adjusted life years associated with PUD than women in all years from 1990 to 2019 (2). In 2019, there were 3.92 million prevalent cases of PUD in females compared to 4.17 million in males, and the proportion of prevalent cases between males and females was 1:0.94 (2). Possible reasons for the higher prevalence of PUD in males may involve biological and physiological differences between males and females, such as hormonal differences (i.e., the protective effects of estrogen in women) affecting the development of ulcers (33–35).

Another interesting finding is that hyperuricemia was associated with a low prevalence of self-reported PUD in the male participants (OR = 0.890; 95% CI = 0.837–0.947; *p* < 0.001). This finding provides evidence supporting the protective effect of hyperuricemia against PUD in males. Previous studies have reported a link between hyperuricemia with an increased risk of several systemic diseases, notably gout and various cardiovascular issues (such as hypertension, heart structure and function), dyslipidemia, chronic kidney disease, thyroid disorders, and metabolic syndrome (15–19). Whereas, another study discussing the relationship between uric acid and osteoporosis suggested that hyperuricemia could have a protective effect against neurodegenerative diseases and may reduce the risk of bone fractures by affecting bone mineral density (24). In the narrative review by Otani et al. (36), the authors investigate the complex role of uric acid in neurological disorders. It details how uric acid’s antioxidant properties might confer neuroprotection by scavenging free radicals and inhibiting lipid peroxidation (36). Notably, the review presents evidence indicating that higher uric acid levels might be linked to a decreased risk and slower progression of Parkinson’s disease (36). High uric acid levels might have contributed to the development of higher intelligence and better neurological health in humans by providing significant antioxidant capacity (36, 37). In a randomized, double-blind, placebo-controlled study involving 24 participants, the effects of intravenously administered uric acid on endothelial function were explored (38). Each participant received 1,000 mg of uric acid, vitamin C, vehicle alone, or saline across separate sessions. Forearm blood flow responses to acetylcholine and sodium nitroprusside were measured using venous occlusion plethysmography. The study found that uric acid, like vitamin C, improved endothelial responses to acetylcholine in diabetics and smokers, suggesting that high levels of uric acid could have protective cardiovascular effects in conditions associated with increased oxidative stress (38). In addition, a study conducted in Taiwan of 1,166 patients hospitalized for ischemic stroke found that higher serum uric acid levels were correlated with better neurological outcomes in male patients but not in female patients, especially in those with the large-artery atherosclerosis stroke subtype (22). The study suggested that serum uric acid level might have a neuroprotective role due to its antioxidant properties, which could be particularly beneficial in the context of oxidative stress during acute ischemic stroke (22). In addition, an animal study observed a relationship between the intestinal tract in mice and uric acid (39). In that study, mice were given inosinic acid to create high and moderate levels of serum uric acid. When the mice were given indomethacin, a medication that typically causes enteropathy, those with higher uric acid levels showed less damage and reduced intestinal reactive oxygen species. The authors concluded that elevated levels of uric acid in the mice seemed to protect their intestines from damage (39). It is also mentioned that uric acid significantly influences gut microbiota composition, which plays a key role in its protective effects against enteropathy. Studies have demonstrated that mice with elevated uric acid levels exhibit richer α-diversity and distinct β-diversity in their gut microbiota compared to controls. This more diverse microbiota potentially enhances the gut’s defense against pathogenic bacteria and supports intestinal integrity (39). Hyperuricemia might reduce the risk of PUD due to its strong antioxidant properties, which can mitigate oxidative stress—an underlying factor in the pathogenesis of PUD (39). Oxidative stress can damage gastric mucosal linings (39), and the antioxidant capability of uric acid may help protect against this damage. The study by Wada et al. (39) provides significant insights into the potential protective mechanisms of uric acid against indomethacin-induced enteropathy, particularly focusing on its role within the intestinal lumen. Their findings suggest that luminal uric acid may protect against gastrointestinal damage through antioxidant properties of uric acid and modulation of gut microbiota. Furthermore, the transplantation of fecal microbiota from mice with high uric acid levels into other mice ameliorated indomethacin-induced enteropathy, underscoring the significant role of microbiota in mediating uric acid’s protective effects (39). These findings may partially explain our finding that hyperuricemia was associated with a low prevalence of self-reported PUD in the male participants.

We also found that hyperuricemia was associated with a low prevalence of self-reported PUD in the male participants (OR = 0.890; 95% CI = 0.837–0.947; *p* < 0.001), whereas this association was not found in the female participants (*p* = 0.139). The absence of a relationship between high uric acid levels and PUD in women might be due to sex differences in uric acid metabolism and its biological effects (40). Sex differences have been observed in many diseases, including cancer, cardiovascular and liver diseases, and these differences can have a major influence on the clinical presentation, disease progression, and response to treatment (9). The mechanism behind sex differences and sexual dimorphism is considered to be linked to sex hormones (41). Another possible reason may be that women have larger subcutaneous fat stores than men, providing better lipid storage and starvation resistance. Female mitochondria exhibit higher functional capacity and resistance to oxidative damage, reducing the transmission of metabolic disorders (42). Sex differences have also been noted in immune responses, with females exhibiting stronger T cell and humoral immune responses compared to males in adaptive immunity (43). Hormonal differences, especially the role of estrogen, may influence how uric acid affects the body (44), potentially altering the risk and severity of PUD. Moreover, women typically have lower uric acid levels than men due to hormonal regulation and renal excretion, which could account for the lack of association (45). Further research is required to delineate these mechanisms more clearly.

The key strength of this population-based investigation is that we included a large study cohort of adults living in the community. Several limitations should also be mentioned. First, as this was a cross-sectional study, we were unable to evaluate the duration of illness, and consequently we were unable to evaluate causal relationships between hyperuricemia and PUD. Longitudinal studies to evaluate the risk of incident PUD are warranted. Second, the presence of PUD was assessed using self-reported questionnaires, and therefore the severity and type of PUD were unknown. Nevertheless, Wu et al. (46) reported a moderate concordance between claims records and self-reported renal diseases in Taiwan. Third, some medications may influence the value of uric acid, and result in PUD were lacking in TWB, which may influence our analysis. Finally, the Chinese ethnicity of our participants may limit the applicability of our findings to other groups.

## Conclusion

In conclusion, we identified a higher prevalence of self-reported PUD in the male participants than in the female participants. Furthermore, we found a significant interaction between sex and hyperuricemia on self-reported PUD. Hyperuricemia was associated with a low prevalence of self-reported PUD in the male participants but not in the female participants in this large Taiwanese population study. Further studies are needed to clarify the mechanisms behind these observations and verify the potential protective role of hyperuricemia on the development of PUD.

## Data availability statement

The raw data supporting the conclusions of this article will be made available by the authors, without undue reservation.

## Ethics statement

The studies involving humans were approved by the Institutional Review Board of Kaohsiung Medical University Hospital (protocol code KMU-HIRB-E(I)-20210058 and 8 April 2021 approval). The studies were conducted in accordance with the local legislation and institutional requirements. The participants provided their written informed consent to participate in this study.

## Author contributions

C-SY: Writing – original draft. J-HG: Conceptualization, Data curation, Methodology, Writing – review & editing. P-YW: Conceptualization, Data curation, Writing – review & editing. J-CH: Conceptualization, Data curation, Writing – review & editing. H-MH: Conceptualization, Data curation, Writing – review & editing. S-CC: Conceptualization, Data curation, Formal analysis, Methodology, Writing – review & editing. C-HK: Conceptualization, Data curation, Supervision, Writing – review & editing.
